# Rotator cable in pathological shoulders: comparison with normal anatomy in a cadaveric study

**DOI:** 10.1007/s12565-018-0447-9

**Published:** 2018-07-10

**Authors:** Michał Tomasz Podgórski, Łukasz Olewnik, Piotr Grzelak, Michał Polguj, Mirosław Topol

**Affiliations:** Instytut Centrum Zdrowia Matki Polki w Łodzi, ul. Rzgowska 281/289, 93-338 Lodz, Poland

**Keywords:** Rotator cable, Glenohumeral joint, Joint capsule, Ligamentum semicirculare, Rotator cuff injury

## Abstract

The rotator cable is a semicircular thickening of the glenohumeral joint capsule. It travels between tubercles of the humerus and interweaves with the supra- and infraspinatus muscle tendons. The rotator cable anchors these tendons to the tubercles, playing the role of a suspension bridge. However, little is known about the modifications of this cable that result from pathologies to the rotator cuff tendons. Thus, we aim to compare the morphology of the normal rotator cable with cables in specimens with rotator cuff injuries. The glenohumeral joint was dissected in 30 cadaveric shoulders. The supra-, infraspinatus and teres minor muscles were inspected for injuries and the rotator cable was visualised. The cables course was determined and the width, length and thickness were measured. The rotator cable was found present in all cadavers dissected. In three specimens there was a partial injury of the supraspinatus tendon (two from capsular side and one from bursal side). The rotator cable was thickened in the cases of capsular tears. In another two specimens the supraspinatus and infraspinatus muscles were torn completely and in these cases the rotator cable was blended with retracted stumps and elongated to the level of the glenoid rim. The rotator cable creates a functional complex with the supra- and infrasinatus muscles. The morphology of the cable differs in cases of rotator cuff injury.

## Introduction

Distal parts of the supra- and infraspinatus muscles are tightly bonded with the glenohumeral joint capsule (Czyrny 2012; Pouliart et al. 2007). In this region there is a crescent of fibres perpendicular to the axis of tendons that runs from the intertubercular groove to the posterior aspect of the greater tubercle. This structure has been described by many authors by different names. It is most well known as the “rotator cable” (Burkhart et al. 1993) or the “ligamentum semicirculare humeri” (Kolts et al. 2000). Others have described it as the “circular fibres system” (Gohlke et al. 1994) or the “transverse band” (Clark and Harryman 1992). Despite the varying nomenclature, it is uniformly known to be part of a functional complex with spinatus muscles (Rahu et al. 2016) and plays a role similar to a cable of a suspension bridge. Thus, according to Burkhart et al. (1993) it transfers the forces of supra- and infraspinatus muscles to the humerus in particular cases of rotator cuff tears. Upon detailed analysis of the literature, however, we found no comparison of normal and pathological anatomy of the rotator cable.

During standard dissection of the glenohumeral joint capsule, we came across a specimen with a total tear of the supraspinatus and infraspinatus muscle tendons. However, muscle bellies were not atrophied and they were anchored to the humerus by a thick fibrous band, possibly the rotator cable. This investigation, inspired by this finding, aims to compare normal and pathologically adapted rotator cables.

## Materials and methods

Thirty isolated, cadaveric shoulders (15 right and 15 left), embalmed in 10%-formalin solution, were included in this study. They were derived from white, adult deceased donors (19 women and 11 men, mean age 57 ± 17 years), who voluntarily signed a donation form in life. Signs of former treatment (e.g. scars, sutures) were exclusion criteria. All limbs were dissected according to the following protocol.

The skin and subcutaneous tissue were removed, followed by the detachment of the deltoid and trapezius muscles from the clavicle and scapulae. The clavicle was then excised from the acromioclavicular joint. Next, the acromion was sawed off at its base and retracted with the coracoacromial ligament anteriorly. Then the subacromial/subdeltoid bursa was excised and all the muscles of the rotator cuff, now visible, were inspected for injuries. Then the teres minor, infra- and supraspinatus muscles were bluntly detached from their medial attachment to the infra- and supraspinatus fossa, respectively. Vessels and nerves supplying muscles were cut through. Afterwards, tendons of muscles were separated from the joint capsule up to the anatomic neck of the humerus to visualize the whole extent of the rotator cable. Then the width of the anterior, middle, and posterior portions were measured. An elastic metal wire was extended along the halfwidth of the cable and measured to obtain the length of the entire cable. Afterwards, the cable was cut perpendicular to its course, at the level of supraglenoid tubercle (approximately at half of its length), to evaluate its thickness. Then the distance from the supraglenoid tubercle and the anatomic neck of the humerus was measured to the closest border of the rotator cable. All measurements were obtained with the limb in anatomical position (scapulae directed about 30° to the back, limb laying straight with hand supine). Measurements were performed twice with an electronic digital calliper (Mitutoyo Company, Kawasaki-shi, Kanagawa, Japan) by two independent investigators. During analysis the mean results of these two measurements were used. All shoulders with pathologies of the rotator cuff were studied in detail and lesions were measured. The other measurements of the rotator cable were performed in the same manner as described above. Study protocol, including cadavers procurement, was approved by the Local Bioethical Committee (protocol number: RNN/241/16/KE).

## Results

### Normal anatomy

**Fig. 1:**
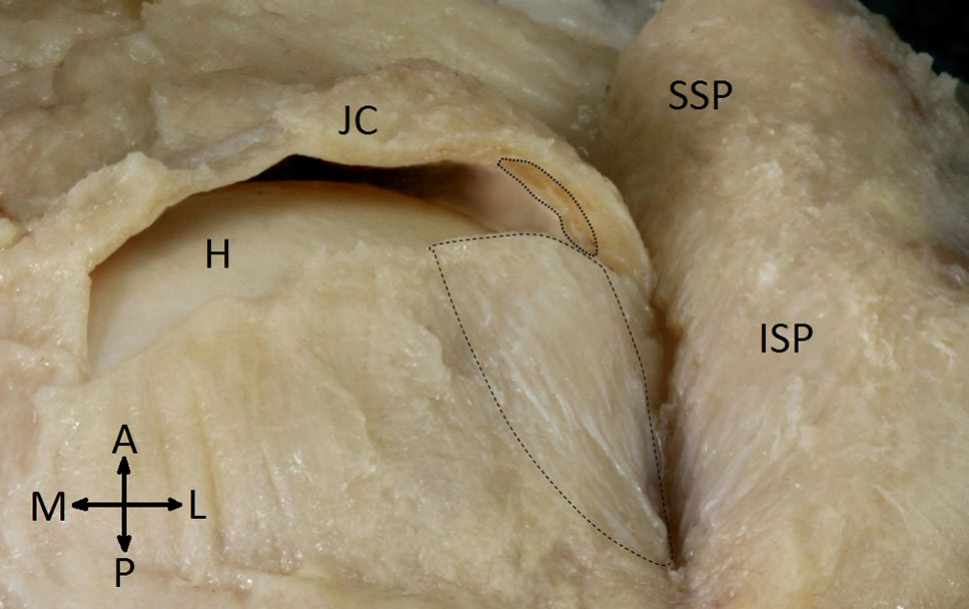
A normal rotator cable (*dashed line*) running within the joint capsule and its cross-section (*dotted line*) at the point of coronal joint capsule transsection. *H* Head of humerus, *SSP* tendon of supraspinatus muscle, *ISP* tendon of infraspinatus muscle, *JC* joint capsule, *A* anterior, *P* posterior, *M* mediale, *L* lateral

In 25 of the 30 specimens there were no rotator cuff ruptures. In all these cases the normal rotator cable was a crescent of bundles running anterior to posterior from the top of the intertubercular groove to the posterior surface of the greater tubercle (Fig. 1). The dimensions are presented in Table 1.

**Table 1:** Dimensions of the rotator cable in glenohumeral joints with normal and injured rotator cuff

Condition of rotator cuff tendon	Width (mm)			Length (mm)	Thickness (mm)	Distance (mm)	
	Post.	Mid.	Ant.			To the supraglenoid tubercle	To the anatomic neck of humerus
Normal [mean (SD)] (*n* = 25)	8.79 (1.17)	8.95 (1.54)	7.76 (1.28)	56.42 (10.13)	1.56 (0.70)	20.68 (7.29)	7.06 (5.66)
Bursal	10.54	8.89	8.63	62.89	1.62	15.67	8.35
Partial tear (*n* = 3)							
Capsular (case1)	8.15	6.91	9.2	74	2.43	16	8.6
Capsular (case 2)	11.91	8.53	6.61	56.11	3.71	17.08	8.21
Total tear (*n* = 2)							
Case 1	10.58	9.91	7.62	118.55	3.18	In contact	36.21
Case 2	10.13	10.14	9.68	95.36	1.92	In contact	31.36

*Post.* posterior part of the rotator cable, *Mid.* middle part of the rotator cable, *Ant.* anterior part of the rotator cable

### Partial rotator cuff tear

**Fig. 2:**
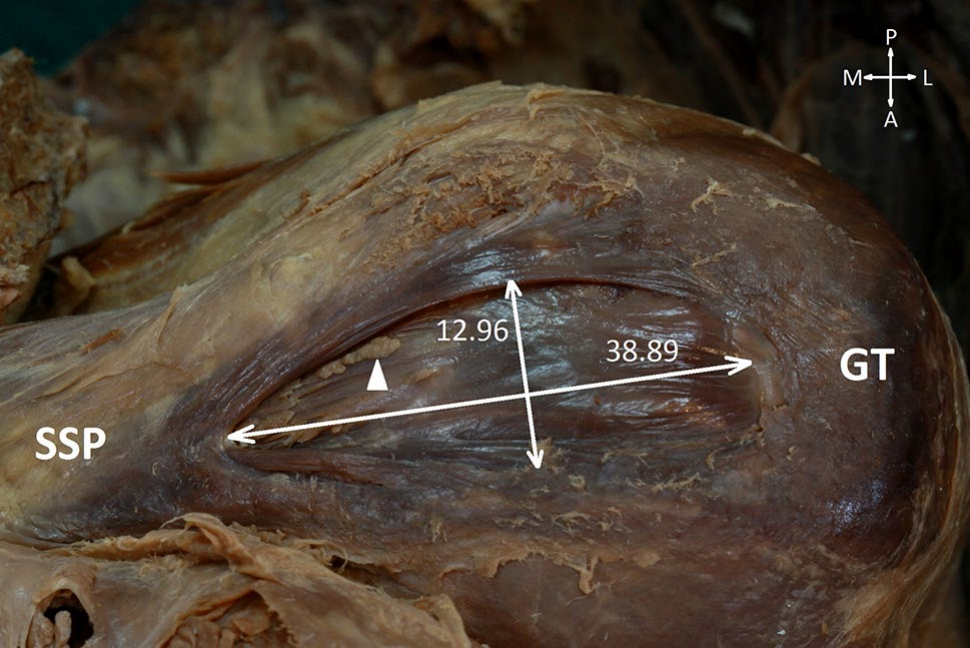
Partial tear of the supraspinatus tendon occurring from the bursal side. Dimensions of lesion are presented in millimetres. *SSP* Supraspinatus muscle, *GT* greater tubercle, *A* anterior, *P* posterior, *M* mediale, *L* lateral,* white triangle* synovial folds

We observed three cases of partial supraspinatus tendon tear concerning the right limb. In one case, the partial tear prevailed from the bursal side (characteristic of lesion is presented in Fig. 2). In the remaining two cases the capsular side was ruptured. The anterior to posterior and medial to lateral dimensions of the supraspinatus lesions were 12.1 × 6.7 mm and 9.8 × 8.5 mm for cases one and two, respectively. In all three specimens, the edges of the lesions were smooth and small fringes of synovial folds were present. These chronic/degenerative changes indicate that the injuries occurred before death.

In the specimen with the bursal tear, the rotator cable had a normal appearance and typical dimensions (Table 1). However, in the shoulders where the capsular side was ruptured, the cables were thicker, but the other dimensions were standard (Table 1).

### Total rotator cuff tear

**Fig. 3:**
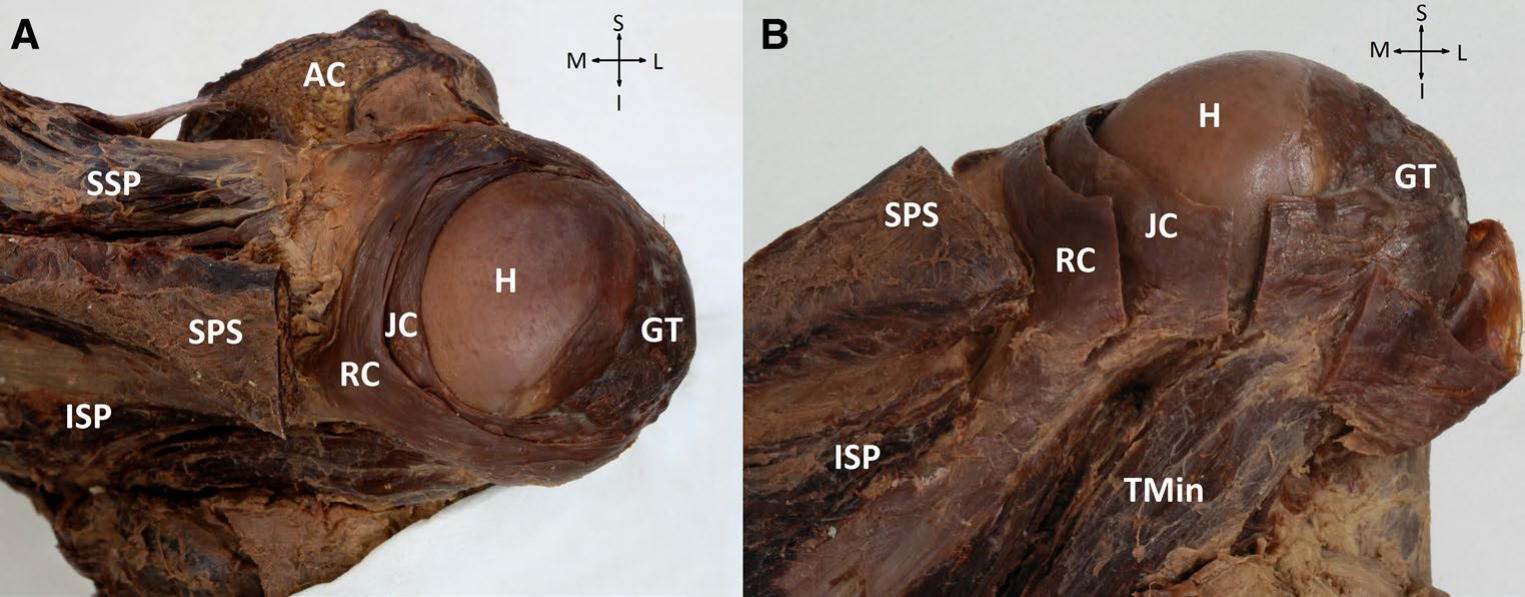
**a** Posterior aspect and **b** postero-inferior of the glenohumeral joint with total tear of the supraspinatu and infraspinatus muscles (case 1 of total tendon tear). *RC* Rotator cable, *JC* joint capsule, *SSP* supraspinatus muscle, *ISP* infraspinatus muscle, *Tmin* teres minor muscle, *H* head of humerus, *GT* greater tubercle, *SPS* spine of the scapula, *AC* acromion, *S* superio, *I* inferior, *M* mediale; *L* lateral

We observed two cases of total rotator cuff tears concerning supra- and infraspinatus muscles (one on the right and one on the left side) (Fig. 3). Muscle bellies had normal appearances. In both cases, retracted stumps of the muscles were fused together at the base of the spine of the scapulae. At the same place they were also blended with a semicircular band of fibres attaching to the posterior surface of the greater tubercle, going round to the middle, and then travelling laterally to attach at the top of the intertubercular grove. This structure was identified as an elongated and medially retracted rotator cable. Under the cable there was another band-like structure fused with the rim of the glenoid, therefore we assumed that it was a conglomerate of degenerated glenohumeral joint capsule and glenoid labrum.

## Discussion

Based on the difficulty of its dissection, we can understand that researchers have varying anatomical theories about the rotator cable. The glenohumeral joint capsule is a complex of entangled fibrous bands that blend together supporting passive joint stability (Clark and Harryman 1992; Czyrny 2012; Gohlke et al. 1994; Kask et al. 2008; Kolts et al. 2000; Nimura et al. 2012; Pouliart et al. 2007; Pouliart and Gagey 2006). Most of the concerns address the anterior part of the cable that ends in the region of the rotator interval, interweaving with the coracohumeral and superior glenohumeral ligaments (Choo et al. 2014; Kask et al. 2008; Kolts et al. 2000; Pouliart et al. 2007). Embryological studies report only that the development of the glenohumeral joint capsule occurs during Carnegie stages 22–23 (Aboul-Mahasen and Sadek 2002; Fealy et al. 2000; Hita-Contreras et al. 2017). However, as it has been reported in macroscopic (Burkhart et al. 1993; Rahu et al. 2016) and microscopic (Fallon et al. 2002) studies, and as we confirmed in normal and pathological specimens, rotator cables form a functional complex with the supra- and infraspinatus muscles. Moreover, our dimensions of the cables were comparable with data from the recent MRI (Gyftopoulos et al 2013) and ultrasound (Orlandi et al. 2012; Sconfienza et al. 2012) studies where only the dimensions of the cable, not the complex with the spinatus tendons, were analysed. Finally, our investigations also suggests that the rotator cable may become thicker with the development of degenerative changes to the rotator cuff, which is in accordance with the MRI study of Choo et al. (2014).

In addition, the results of our study allow us to hypothesize that the morphological changes in the rotator cable depend on the type of injury sustained. For example, partial capsular tears of spinatus tendons may separate them from the cable eliminating (partially or totally) its role as a “suspension bridge line”. On the other hand, when the total rotator cable tears, but the integrity of the rotator cable is preserved, the cable may transfer the pressure from the spinatus muscles to the humerus, protecting muscles from complete atrophy and preventing a severe weakness of external rotation. This observation might also support why elderly people with spinatus muscle tears present few symptoms and maintain sufficient function of their glenohumeral joints (Halder et al. 2002).

Another implication from this work may concern diagnostic imaging. The appearance of the rotator cable has been described by ultrasound (Morag et al. 2006; Orlandi et al. 2012; Sconfienza et al. 2012) and MRI (Gyftopoulos et al. 2013; Kask et al. 2008) previously. However, depending on co-occurring pathologies and applied technique, the cable may only be visible in 0–74% of patients by MRI. Recently, Choo et al. (2014), applying indirect MR arthrography, was able to visualise the rotator cable in 100% of patients with normal and partially injured rotator cuff tendons. However in patients with total tears, the rotator cables were only visible in 76% of patients. Distinguishing the rotator cable from the retracted lateral edge of the rotator cuff tear was found to be difficult (Choo et al. 2014). Our study may explain this difficulty: during a total tear, the rotator cable elongates aligning close to the ridged joints capsule and labrum, fusing with the torn tendon stumps.

Our work might also be important from an orthopaedic point of view. Rotator cuff lesions destabilize the glenohumeral joint not only due to the lack of active stabilizers, but also by passively interfering with the functions of ligaments. However, this concerns only lesions in the humeral region, not close to the glenoid (Pouliart and Gagey 2006). This is probably a result of the interdigitating of cuff tendons and capsules on the humeral side of the tendons. This hypothesis is supported by our observations of total rotator cuff tears, where the distal detachment of the rotator tendons tears the joint capsule and pulls the rotator cable medially. As the rotator cable forms a complex with other passive stabilizers of the glenohumeral joint, (superior glenohumeral ligament, coracohumeral ligament) (Kask et al. 2008) further joint stability may be impaired.

The main limitation of this study is the quantity of pathologically changed rotator cuffs examined. However, the presented cases are highly suggestive and generated interesting hypotheses that should be tested in further anatomical and clinical studies. Secondly, specimens presenting pathologies were exsiccated. Due to contraction of soft tissues this might bias measurements. Nevertheless, these measurements were used only for descriptive purpose and no for statistical calculations. Thus, we assume that despite dehydration, our conception concerning the role of the rotator cable is true.

## Conclusion

We presented anatomical descriptions of normal and pathologically modified rotator cables. Our observations suggest that the location and extent of a rotator cuff tear may affect the morphology of the rotator cable.
